# Stimulation of Ideas through Compound-Based Bibliometrics: Counting and Mapping Chemical Compounds for Analyzing Research Topics in Chemistry, Physics, and Materials Science

**DOI:** 10.1002/open.201200029

**Published:** 2012-10-25

**Authors:** Andreas Barth, Werner Marx

**Affiliations:** [a] Fachinformationszentrum (FIZ) Karlsruhe, Hermann-von-Helmholtz-Platz 176344 Eggenstein-Leopoldshafen (Germany); [b] Max Planck Institute for Solid State Research, Heisenbergstr. 170569 Stuttgart (Germany)

**Keywords:** information retrieval, quasicrystals, rare earths, scientometrics, superconductors

## Abstract

Counting compounds (rather than papers or citations) offers a new perspective for quantitative analyses of research activities. First of all, we can precisely define (compound-related) research topics and access the corresponding publications (scientific papers as well as patents) as a measure of research activity. We can also establish the time evolution of the publications dealing with specific compounds or compound classes. Moreover, the mapping of compounds by establishing compound-based landscapes has some potential to visualize the compound basis of research topics for further research activities. We have analyzed the rare earth compounds to give an example of a broad compound class. We present the number of the currently existing compounds and of the corresponding publications as well as the time evolution of the papers and patents. Furthermore, we have analyzed the rare earth cuprates (copper oxides) as an example of a narrower compound class to demonstrate the potential of mapping compounds by compound-based landscapes. We have quantified the various element combinations of the existing compounds and revealed all element combinations not yet realized in the synthesis within this compound class. Finally, we have analyzed the quasicrystal compound category as an example of a compound class that is not defined by a specific element combination or a molecular structure.

## Introduction

Since large volumes of data have become publicly available in the last years *big data science* has gained a lot of interest as a new topic of research.^1^ Big data science integrates science and information technology in completely new ways, and it also addresses many issues encountered outside of scientific research such as copyright protection and the organization of data (e.g., workflow, data sharing). It comprises the infrastructure to store, manage, and retrieve large amounts of data, the tools for analysis and visualization,^2^ as well as the corresponding applications in scientific disciplines. Several new terms have been proposed to describe the new field of big data research, for example, grid, e-science, or cyberinfrastructure. In general, big data science is seen as a huge challenge to science as well as to society as a whole.^3^

In chemistry, a number of papers have been published on new applications of big data science.^4^–^6^ In most cases the methods and technologies are applied to publicly available open-source databases such as PubChem,^7^ which are limited in scope and size. Some applications focus on special areas within chemistry, and hence it is possible to work with subject-specific chemical databases such as the Cambridge Structural Database (CSD).^8^ However, in order to perform a complete analysis of chemical compounds, it is necessary to work with the most comprehensive databases in the field, that is, the compound file (Registry) and literature file (CAplus) from Chemical Abstracts Service (CAS).^9^

In this paper, we have extended the bibliometric method to chemical compounds and, thus, defined compound-based (chemical) bibliometrics as a new research field. The method can be applied to analyze large amounts of compounds in combination with the corresponding chemical concepts, to identify gaps in research, and hence open the door to new research in well-described compound-based areas. As applications of our method, we have chosen three examples from inorganic chemistry: rare earth compounds, rare earth cuprates, and quasicrystals. Although our examples are from inorganic chemistry, the method can easily be extended to organic chemistry or to biochemistry.

Bibliometrics or the broader term, scientometrics—both terms are often used synonymously—can be characterized as the discipline that treats science quantitatively. Publication and citation numbers are the most important items that have become the basis of bibliometric indicators for research evaluation purposes. In many disciplines, particularly in chemistry, physics, and materials science, chemical compounds (substances) play a major role. We may speak of *compound culture*^10^ if a research field is based on a single compound (material) or a compound class (a compound family with a specific combination of elements or containing a specific substructure). A formal comparison uncovers a pronounced analogy between bibliographic references (on which citation counts are based) and chemical compounds mentioned in the scientific literature: Both items can be seen as concept symbols^11^ that are coded and linked within the relevant databases, and they can be consulted for quantitative analyses. Citation counts are increasingly used for research evaluation as a measure of the impact of scientific papers.^12^ Similarly, the number of compounds within a given compound class as well as the number of the corresponding publications (papers as well as patents) can be seen as a measure of research activity within the relevant research field. The number of patents reveals the amount of application of research activities.

Counting compounds rather than publications or citations opens a new perspective to quantitative analysis of research activities. We can precisely define (compound-related) research topics and easily access and refine the corresponding publications. Furthermore, we can establish the time evolution of the publications dealing with specific compounds or compound classes. Moreover, the mapping of compounds by establishing compound-based landscapes has some potential to visualize the compound basis of research topics. Particularly, in the case of inorganic chemistry and materials science, the mapping of element combinations realized in the synthesis of the compounds within a specific compound class reveals the appearance of hot topics (with many existing compounds and papers) and also the existence of white patches within compound landscapes (i.e., element combinations not yet realized in the synthesis of compounds). The compound-based mapping concept can easily be extended to organic chemistry by searching molecular substructures rather than element combinations. Chemists obtain an overview and use the results as a basis for further research.

## Methodology

The databases offered by Chemical Abstracts Service (CAS), a division of the American Chemical Society (ACS), are the most powerful and sophisticated source of compound-related literature (either papers or patents) in the fields of chemistry, materials science, and physics.^13^ The CAS literature file (Chemical Abstracts Plus, CAplus) covers both papers and patents published since around 1900. The CAS compound file (Registry) contains all chemical species mentioned within chemistry and related research fields. These items (compounds and papers) are called database documents or records. The search options and additional functions available through the online service, the Scientific and Technical Information Network (STN International),^14^ have made it possible to perform extensive bibliometric studies.^15^ Alternatively, CAS offers SciFinder as a research discovery tool that provides scientists with web access to explore the CAS files. The competent use of such databases and search systems, however, requires some experience and awareness of possibilities and pitfalls.

The CAS Registry file contains records for all chemical compounds identified and registered by the CAS Registry system since around 1906. All compound records are associated with a unique CAS Registry number. The records include various compound information, in particular CA index names, molecular formulas, structure diagrams, and the number of references in the CAS literature file CAplus. All of this information is displayable and searchable on STN.

Using the CAS Registry file and counting compounds brings about several advantages, which are summarized in Table 1. The most important features in searching chemical information are: 1) Compounds are unambiguously coded by their Registry numbers, and 2) compounds can be searched by using the sophisticated search functionalities of the Registry file (e.g., by searching their standard chemical names, molecular formulas, and structure diagrams). Searching only the common compound names within titles or abstracts in the CAS literature file (or in any other literature file) is an incomplete and unspecific search method in chemistry.

**Table 1:** Comparison of citations and compounds as bibliometric items

Citations	Compounds
Incomplete (social act)	Complete (intellectually indexed)
Error-prone (mutations)	Unambiguous, precise, specific
Obliteration	No obliteration
Difficult to categorize	Easy to categorize
Access through papers	Access through molecular formulas and structures, reactions, bio-sequences

The advantages of compounds as bibliometric items imply a high potential for various applications and goals. The in-depth analysis of large compound classes and the corresponding literature is most important to researchers. The analysis is mainly based on the compound-specific selection possibilities (searching element and/or periodic group combinations, searching structure or substructure diagrams) of the Registry file. It is necessary to distinguish two cases of searching:

Case A: The compound class is clearly defined in terms of a specific combination of chemical elements or a specific molecular substructure diagram.
Case B: The compound class can only be defined by a general name. In this case we have to locate the compound set through a stepwise iteration of searches in the literature file based on text searching.

The potential of compound-based bibliometrics is illustrated by three typical examples. As examples of case A compound classes, we have first analyzed rare earth compounds as a whole. In addition, we have analyzed the rare earth cuprates (copper oxides), which are most relevant to the research field of high-temperature superconductivity. As an example of a case B compound class, we have analyzed the compound basis of the so-called quasicrystals.

**Figure 1:**
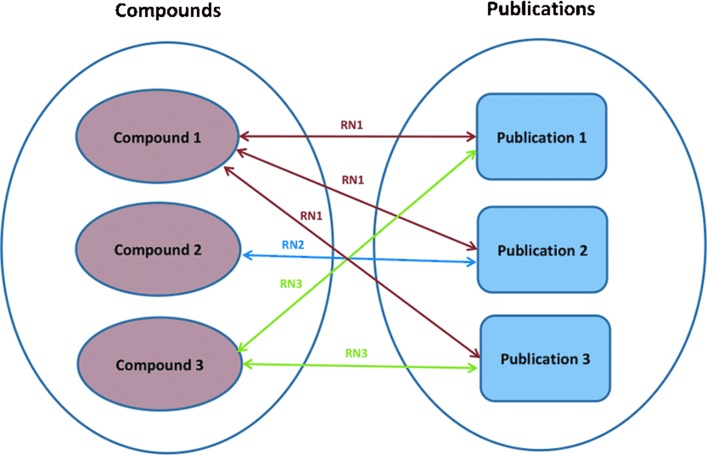
Illustration of the correspondence between the two separate data pools of chemical compounds and the corresponding publications as given in the architecture of the CAS files. In addition, the file crossover through the Registry numbers (RN1–RN3) is shown.

Whenever we analyze compound sets and the corresponding literature, we have to combine compound and literature information that correspond to two different concepts and, hence, reside in two different databases. The searches involve file crossover from the compound file to the literature file and/or vice versa. The two separate data pools of the chemical compounds and the corresponding literature information as well as the transfer of Registry numbers are illustrated in Figure 1.

The separation of compounds and literature by CAS and the searching in two different databases implies a somewhat unusual processing compared to standard literature searching. The two different search steps for case A searches are described in Table 2.

**Table 2:** The different search steps for a case A analysis

No.	Step[a]	Action	Database
1	Create an answer set of all chemical compounds (C1) belonging to a specific compound class	Search for element combinations or molecular substructure diagrams	Registry
			
2	Create an answer set of either all publications (P1) corresponding to the compound class (C1) or only of the publications relevant for a specific research topic (P2)	a) Transfer the RN’s to the literature file	CAplus
		b) Search for the RN’s linked with a specific text term or keyword (using LINK proximity)	

[a] C1 comprises all compounds according to the original definition of the compound class. P1 contains all corresponding publications (papers and patents) and P2 only contains the publications corresponding to a specific topic. RN: Registry number.

## Rare earth compounds

We have analyzed the rare earth compounds as a whole to give an example of a broad and voluminous case A compound class (with regard to both the overall number of compounds and the number of the corresponding publications). All compounds containing one of the various rare earth elements have been selected, rather than any specific element combination as performed in the subsequent example. Our aim is to quantify the element-specific amount of existing compounds and the corresponding literature published as papers and patents.

Rare earth elements have become a topic of public discussion, since it is known that these elements are of high importance to a number of essential new technologies and applications. Selected usages include high-temperature superconductors, catalysts, computer technology, lasers, light-emitting diodes (LEDs), cell phones, permanent magnets, green energy, and many others. Although these elements are not as rare as the name suggests, the worldwide resources are limited, and they are only found in viable amounts in some regions of the world. Due to their physical and chemical properties, these elements are typically dispersed, and the production is rather expensive.

While public search engines deliver impressively large hit numbers, the following questions arise. What is a realistic figure for the total number of publications of this subject, and what does this figure actually mean? Is it possible to find the total number of compounds, and how can this be related to the set of publications? What types of compounds are known? In order to obtain answers to these questions, it is necessary to perform more sophisticated searches in high-quality databases.

In the CAS literature file publications on the subject of “rare earth” can be searched in different ways and the results may differ considerably. A search of the phrase “rare earth#” (with wildcard for plural) in the basic index is somewhat similar to the public search engine searches, although much more specific as the search is performed in a field-specific database. The CAS-based search results in 210 833 publications (papers and patents). Alternatively, we can search for the phrase “lanthanide#” (54 563), and for the element names (712 821). Combination of these searches by Boolean OR results in altogether 807 933 publications (date of search: 7. May 2012).

Making use of the CAS Registry file offers a much better method to obtain a precise figure for the number of compounds as well as for the number of scientific papers and patents dealing with these compounds. Rare earth compounds are characterized by the presence of an element of the scandium group (B3) or the lanthanide series (LNTH). The search is based on the molecular formula field and the answer set contains 566 285 compounds (date of search: 7. May 2012). The Registry numbers can be transferred from the compound file to the literature file (and also to other CAS files). As the answer set of the rare earth compounds is very large, it must be divided into smaller sets, which then can be transferred to the literature file. This incremental search of the compound answer set results in a total of 794 683 publications dealing with at least one rare earth compound (including the pure metals).

**Figure 2:**
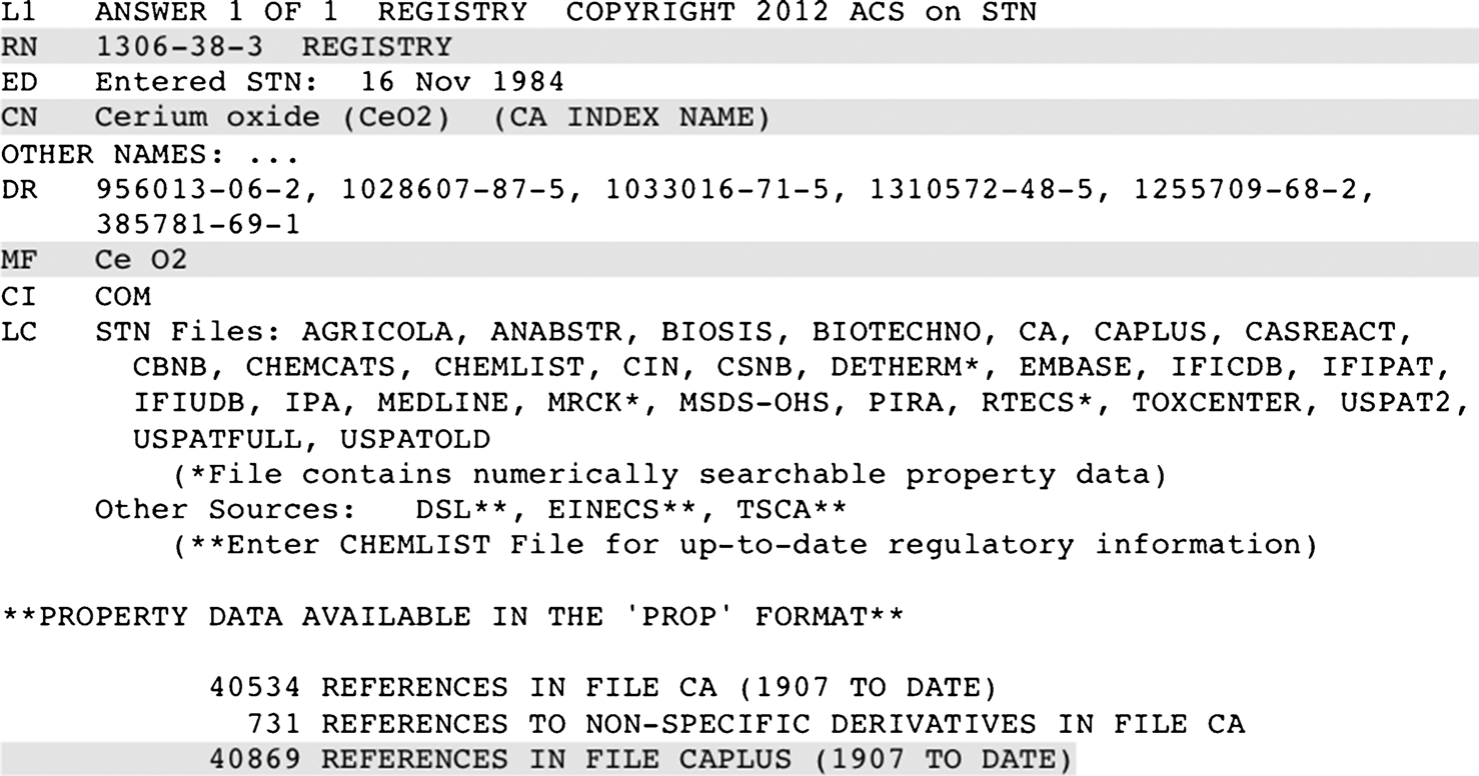
CAS Registry file document of the compound CeO_2_ (ceria). The Registry number (RN) and the chemical name (CN) of the compound are given at the top of the document. The element combination is given in the molecular formula search field (MF). The number of publications (papers and patents) related to the compound and covered by the CAS literature file is given at the bottom. Source: File Registry on STN (date of search: May 7, 2012). The relevant parts of the search procedure and of the database documents are marked in light grey.

**Figure 3:**
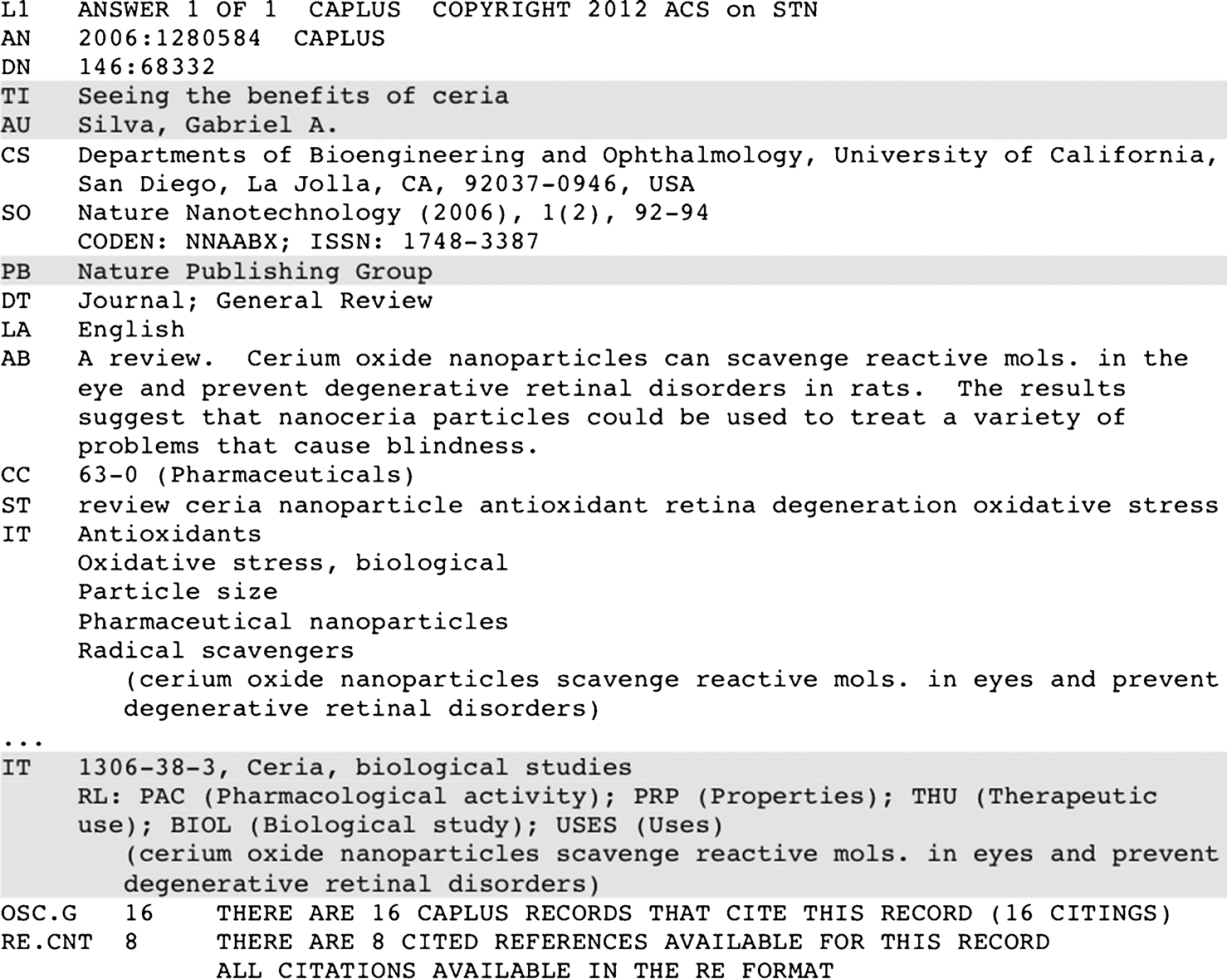
Bibliographic document of a paper referring to CeO_2_ (ceria). The relevant index term (IT) comprises the compound specific Registry number linked with the corresponding keyword terms. Source: File CAplus on STN (date of search: May 7, 2012). The relevant parts of the search procedure and of the database documents are marked in light grey.

Figure 2 shows the Registry file document (compound identification) of the compound CeO_2_ (ceria). It can be seen as an example of a typical species of the rare earth compounds. Note that the relevant parts of the search procedure and of the database documents given in Figure 2–3 and Figure 6 below are marked in light grey.

The publications corresponding to a specific compound class cannot only be searched in total (as above) but also in a more restricting way: The available search functions of the STN search system make it possible to combine compounds (using the corresponding Registry numbers) with index terms (IT, keywords carefully assigned by the database producer) in a way that the compounds and the index terms are interrelated to each other. As a consequence, publications related to specific compounds or compound classes can be selected in conjunction with specific research topics with high completeness and precision. Figure 3 serves as an example of a CAS literature file document of a paper referring to biological studies of CeO_2_ (ceria).

The Registry numbers function as document numbers of the specific compound documents covered by the compound database (see Figure 2) and also as index terms assigned to the relevant bibliographic documents covered by the literature database (see Figure 3). They are the unique link between the compounds and the corresponding publications (see also Figure 1).

**Figure 4:**
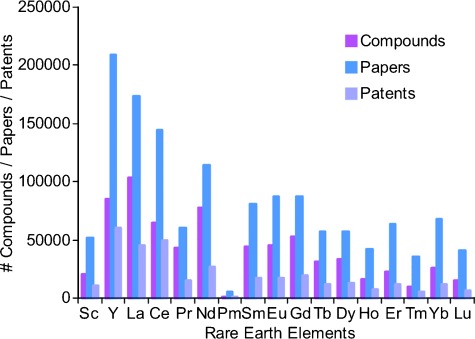
The number of the currently existing compounds, containing specific rare earth elements, as well as the number of the corresponding papers and patents, dealing with rare earth compounds. Source: CAplus and Registry on STN (date of search: May 7, 2012).

The number of the rare earth compounds currently registered by CAS as well as the number of the corresponding publications (papers and patents) provide further interesting information and are given in Figure 4.

**Figure 5:**
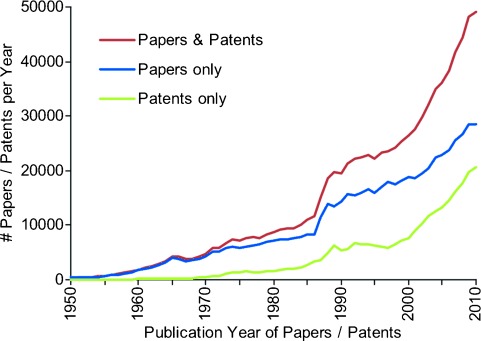
Time curves of the publications (papers and patents) dealing with pure rare earth metals or rare earth compounds based on searching the corresponding Registry numbers in the CAS literature file. Source: CAplus and Registry on STN (date of search: May 7, 2012).

The CAS databases also enable to determine the time curves of publication subsets related to specific compounds or compound classes. Again, the compounds or compound classes are searched in the Registry file and the resulting Registry number sets are then transferred to the literature file, CAplus. A subsequent search in the literature file yields all publications of the original compounds or compound classes. The publications may also be restricted further by combining the original answer set of Registry numbers with additional search terms (keywords) as mentioned above. The number of publications can easily be plotted against the publication year. Figure 5 shows the time curves of the papers and patents dealing with rare earth compounds (including the pure metals).

## Rare earth cuprates

As a second example, we have analyzed the rare earth cuprates (copper oxides) as a more specific case A compound class to demonstrate the potential of mapping compounds, that is, creating compound-based landscapes. Our aim is to quantify the various element combinations of the existing compounds and to reveal all element combinations not yet realized in the synthesis of members of the given compound class.

A new research field around high-temperature superconductors has been established by the unexpected discovery of the superconducting Ba–La–Cu–O system in the year 1986 by Bednorz and Müller.^16^ Since that time, the number of alkaline earth (A2) rare earth (RE) copper oxides (A2 RE cuprates) as well as the amount of the corresponding publications (papers and patents) has strongly increased. A broad class of material has been investigated by the complete tool set of experimental and theoretical solid-state physics. The large number of new superconducting compounds and publications has brought about that scientists working within this research field have increasing problems in overviewing their discipline and staying up-to-date with the new literature.

**Figure 6:**
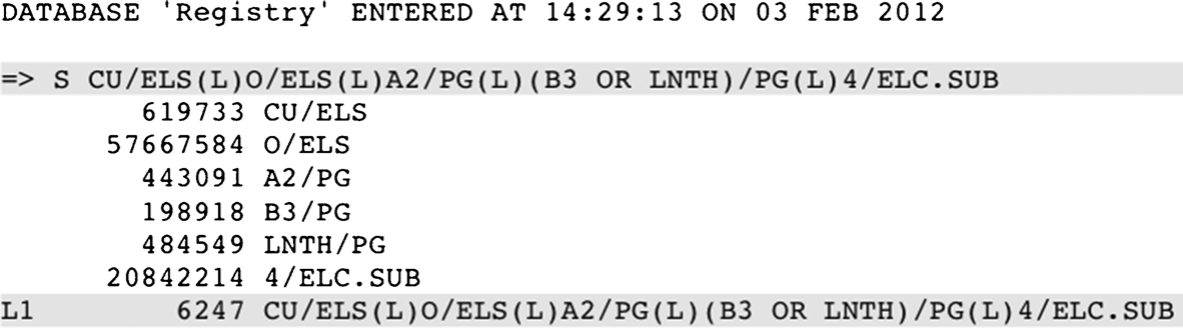
Search query of the search for quaternary A2 RE cuprates in the CAS Registry file. In STN, all answer sets are denoted as subsequent list (L) numbers (there is no formal distinction between answer sets of compounds and publications). B3 and LNTH are database-specific general notations for elements of the scandium group and the lanthanide series. Source: Registry on STN (date of search: February 3, 2012). The relevant parts of the search procedure and of the database documents are marked in light grey.

The CAS Registry file allows easy access to the complete set of compound species, for example of the quaternary A2 RE cuprate compound class registered so far. The selection can be performed by combining the specific elements (Cu and O) and periodic groups (A2 and RE) and restricting the composition of the molecular formulas to exactly four different elements. The search query in the CAS Registry file is shown in Figure 6.

The distribution of publications on the related superconducting compounds is highly skewed: a large fraction of publications refer to a small fraction of compounds. Out of 65 676 publications (58 715 papers and 6 961 patents) dealing with quaternary alkaline earth (A2) rare earth (RE) copper oxides (cuprates), there are 33 405 publications dealing with the specific compound Ba_2_YCu_3_O_7_. On the other hand, a large number of compound species of this quaternary element system is mentioned only a few times or even only once. Note that different preparation methods and analysis accuracies cause a wide range of stoichiometry within one and the same compound class. Differences in stoichiometry above 0.01 result in different compound species (coded by specific Registry numbers) leading to the comparatively high number of species (6247) within the compound class searched here.

**Figure 7:**
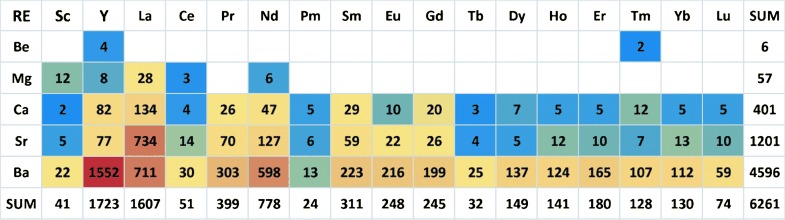
Number of compound species (items with different stoichiometry) as a function of specific combinations of A2 and RE elements of the quaternary A2 RE cuprate compound class. The color differentiation distinguishes the numerous and less numerous existing A2 and RE compound subsets. Source: Registry on STN (date of search: February 3, 2012).

The compound classes selected in the CAS Registry file can be mapped in order to visualize the existing (registered) and non-existing (not yet synthesized) compound species. As an example, the distribution of compound species within the quaternary A2 RE cuprate compound class has been analyzed by determining the number of species as a function of specific combinations of A2 and RE elements. In Figure 7, the occurrence of the A2 main group elements has been plotted against the rare earth (RE) elements. Out of 85 (5×17) possible element combinations of the quaternary system, 58 combinations have been registered by CAS so far. The compound map shown in Figure 7 has been established using Microsoft Excel.

The mapping of element combinations realized up till now in the synthesis of specific combinations of A2 and RE elements of the quaternary A2 RE cuprates reveals the appearance of hot regions with many existing compounds (and usually many corresponding publications) and also the existence of white patches (i.e., element combinations not yet realized in the synthesis of such compounds). The corresponding number of papers and patents could also be included in the map of Figure 7 but has been omitted here to provide a better overview. Experts are certainly aware of the key aspects of activity within their research field but usually miss any quantitative information accessible through this kind of bibliometric method. They can use the results as a basis for further research.^17^

## Quasicrystals

Finally, we have analyzed the quasicrystal compound category as an example of a case B compound class. This class is not defined by a specific element combination or a molecular structure but can only be accessed by searching its general name in the text field of the relevant literature file. There are other materials that are not indexed in the CAS Registry file, for example, the carbon allotropes graphene and carbon nanotubes. The publications related to such material can only be selected by searching the relevant terms within titles, abstracts, and keywords.

In the year 1984, Daniel Shechtman and his co-workers published a paper titled *Metallic Phase with Long Range Orientational Order and No Translational Symmetry*.^18^ Shortly after this, Levine and Steinhardt showed that this phenomenon had already been known to mathematicians and coined the term *quasicrystals* for the new structure type.^19^ The experimental discovery of quasicrystals opened a new research area to crystallography, mathematics, chemistry, materials science, and physics. In 2011, the Royal Swedish Academy of Sciences (Stockholm, Sweden) decided to award the Nobel Prize in Chemistry to Daniel Shechtman (Technion, Israel Institute of Technology, Haifa, Israel) “for the discovery of quasicrystals”.^20^ Quasicrystals are chemical compounds with nonperiodic ordering, making it challenging to preform searches in structured databases. This arises because the search term is very general while there is no way of defining a chemical structure or compound class. In addressing these issues, we will also be able to determine how many papers have been published over time, and which elements/compounds can give rise to quasicrystals, their total number as well as their chemical composition.

Different from the previous cases, the quasicrystal compound class is much too heterogeneous and not clearly defined in terms of elements and substructures. Quasicrystals cannot be searched in the CAS Registry file by combining elements and periodic groups. Therefore, it is necessary to start the search with the general term “quasicrystal” in the literature file and to apply various methods to select information and to toggle between the compound and the literature file. Information searching on such a high level cannot be performed with simple searches in popular search engines. It is necessary to make use of the sophisticated features of STN and the high-quality indexing of the relevant databases to create rich answer sets, which provide additional insight into the publication pattern of this research field and make further analyses of crystal compounds possible.

**Figure 8:**
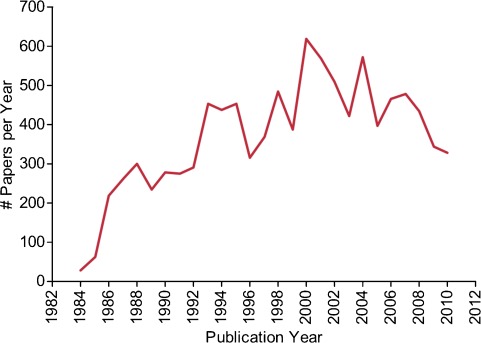
Time curve of quasicrystal papers. Source: CAplus on STN International.

A search in the literature database with various spellings of the term “quasicrystal” yields a total of 10 239 publications within the time period 1984 to 2011 (date of search: 25. October 2011). This comprises all papers (patents play only a minor role here) that contain the term “quasicrystal” in the title or abstract field. The original paper by Shechtman had to be included separately because the term “quasicrystal” was coined later.^18^ It is important to note that this answer set is actually a superset of all kinds of papers, some of which may only refer vaguely to the concept of quasicrystals. The time evolution of the papers is shown in Figure 8. It can be seen that the number of papers increased strongly in 1985 after the publication of the papers by Shechtman^18^ and Steinhardt.^19^ The number of papers reached a peak around the year 2000 with more than 600 papers. Since 2000, the number of papers has slowly decreased.

**Figure 9:**
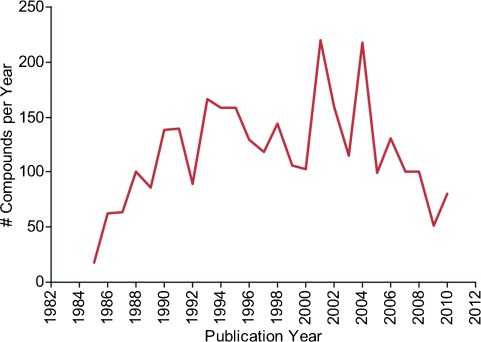
Time curve of quasicrystal compounds. Source: Registry on STN International.

In order to find the complete set of specific quasicrystal compounds, it is necessary to select Registry numbers and perform multiple crossovers between the CAplus and the Registry file. This results in an answer set containing all compounds that are closely related to quasicrystals (3,077), and we may call this the answer set of quasicrystals. A timeline of these compounds is shown in Figure 9.

The different search steps for case B analyses (searches for compound classes that can only be defined by a general name) are summarized in Table 3. It is important to note that in order to obtain the answer sets C2 and P2 it is necessary to search back and forth in both the compound file and the literature file. This process can be continued until the final answer sets are obtained. Both answer sets C2 and P2 may be used for further analyses. The multiple-file crossover is necessary for the following reasons. Firstly, we have to select the compound class in an indirect way using a broad name of a chemically unspecific compound class (quasicrystal) for text searching—rather than searching the molecular formulas in the compound file directly. By this means we select all compounds that are possible candidates for quasicrystals (mentioned within the papers dealing with quasicrystals). However, this answer set contains additional compounds, which are mentioned in the synthesis of quasicrystals but do not form quasicrystals themselves. Secondly, we have to refine the selected compound set by searching only those Registry numbers that are linked with the appropriate keyword (quasicrystal) as index terms within the literature file to make sure that the resulting compounds are actually quasicrystals. Finally, we have to transfer the Registry numbers to the compound file for further analysis using compound-specific search functions. In other words, we have to go a long way to combine the compound-specific and the text-specific search possibilities out of two completely different databases.

**Table 3:** The different search steps for case B analyses

No.	Step[a]	Action	Database
1	Create an answer set of publications (P1) dealing with a broad compound class	a) Search for compound class terms	CAplus
		b) Select the RN’s from the publications	
2	Create an answer set of all chemical compounds (C1) contained in the papers of the publication set (P1)	a) Transfer the RN’s to the compound file	Registry
		b) Search for the RN’s	
3	Create an answer set of all publications containing the RN’s from (C1) which are linked to the original topic	a) Transfer the selected HIT RN’s to the literature file	CAplus
	Create a refined publication set (P2) only containing publications dealing with the selected compounds linked with the name of the broad compound class	b) Search for the RN’s linked with a specific text term or keyword (using LINK proximity)	
		c) Select the HIT RN’s	
4	Create an answer set (C2) of all relevant compounds belonging to the broad compound class	a) Transfer the HIT RN’s to the compound file	Registry
		b) Search for the HIT RN’s	

[a] The initial publication set P1 contains all relevant publications (papers and patents) of a research topic dealing with a broad and unspecific compound class. C1 comprises all compounds mentioned in P1 as potential members of the broad compound class. P2 only contains the papers dealing with the selected compounds linked with the name of the broad compound class (or any other suitable keywords). C2 comprises all relevant compounds belonging to the broad compound class. RN: Registry number.

It is now possible to refine the quasicrystal answer set and to analyze the compound subsets in more detail. We chose alloys as the set of most important compounds from the quasicrystal answer set. Extracting molecular formulas from the alloy subset yields 563 different element combinations (alloy systems) out of 3,031 alloy species with different stoichiometry. The top alloy systems are: 177 alloys of the system Al–Cu–Fe, followed by 155 Al–Mn–Pd, 119 Mg–Y–Zn, 112 Al–Co–Ni, and 101 Ni–Ti–Zr.

The case B searching method illustrated here for quasicrystals could easily be applied to broader and larger research fields related to a multitude of compounds. Furthermore, it would be possible to confine the compounds relevant to the various specialties to certain applications like environmental research (e.g., atmospheric chemistry) or energy research (e.g., batteries, fuel cells).

## Discussion

We have applied bibliometric methods to the field of chemistry in order to reveal and quantify the compound basis and the corresponding scientific literature of three selected research topics (rare earth compounds, rare earth cuprates, and quasicrystals). The compound classes have been confined by selecting chemical elements in the CAS compound file. The compound Registry numbers make precise access to the corresponding literature possible. In order to obtain the compound and literature answer sets, it is sometimes necessary to search back and forth in both the CAS compound file (Registry) and the literature file (CAplus). The number of compounds within a specific compound class as well as the number of the corresponding publications can be seen as a measure of research activity and scientific weight. In the case of inorganic chemistry and materials science, the mapping of element combinations realized in the synthesis of the compounds within a specific compound class reveals the appearance of hot topics and also the existence of white patches. The compound-based bibliometric concept can easily be extended to organic chemistry by searching molecular substructures rather than element combinations, or to biochemistry by searching protein or nucleic sequences.
